# *In Silico* Prediction of Cytochrome P450-Drug Interaction: QSARs for CYP3A4 and CYP2C9

**DOI:** 10.3390/ijms17060914

**Published:** 2016-06-09

**Authors:** Serena Nembri, Francesca Grisoni, Viviana Consonni, Roberto Todeschini

**Affiliations:** Department of Earth and Environmental Sciences, University of Milano-Bicocca, P.za della Scienza 1, 20126 Milano, Italy; s.nembri@campus.unimib.it (S.N.); f.grisoni@campus.unimib.it (F.G.); viviana.consonni@campus.unimib.it (V.C.)

**Keywords:** cytochrome P450, QSAR, CYP2C9, CYP3A4, *in silico*, ADMET

## Abstract

Cytochromes P450 (CYP) are the main actors in the oxidation of xenobiotics and play a crucial role in drug safety, persistence, bioactivation, and drug-drug/food-drug interaction. This work aims to develop Quantitative Structure-Activity Relationship (QSAR) models to predict the drug interaction with two of the most important CYP isoforms, namely 2C9 and 3A4. The presented models are calibrated on 9122 drug-like compounds, using three different modelling approaches and two types of molecular description (classical molecular descriptors and binary fingerprints). For each isoform, three classification models are presented, based on a different approach and with different advantages: (1) a very simple and interpretable classification tree; (2) a local (*k*-Nearest Neighbor) model based classical descriptors and; (3) a model based on a recently proposed local classifier (*N*-Nearest Neighbor) on binary fingerprints. The salient features of the work are (1) the thorough model validation and the applicability domain assessment; (2) the descriptor interpretation, which highlighted the crucial aspects of P450-drug interaction; and (3) the *consensus* aggregation of models, which largely increased the prediction accuracy.

## 1. Introduction

Cytochromes P450 (CYP) are a family of monooxygenase enzymes known for their crucial role in the metabolism of xenobiotics, as they are involved in the oxidation of the majority of compounds [1]. Despite the fact that the human genome encodes up to 57 different CYP genes, only six isoforms are mainly interested in drug metabolism [2,3]. Two of them account for 43% of the metabolism of known drugs: (1) the CYP3A4 isoform, which interacts with more than a half of all clinically used drugs, e.g., large and lipophilic molecules and; (2) the CYP2C9 isoform, which mainly metabolizes NSAIDs (Non-Steroidal Anti-Inflammatory Drugs) and weakly acidic molecules with a hydrogen bond acceptor [4]. These isoforms were the targets of the present work.

The evaluation of the interaction of CYP with chemicals constitutes a fundamental step for drug discovery/design, as well as for toxicity assessment [2,5,6,7]. For this reason, Cytochrome P450 isozymes have been the target of many modelling studies [8,9]. In this context, relevant contributions to the field have been given by Quantitative Structure-Activity Relationship (QSAR) studies, which link the molecular structure, encoded within the so-called molecular descriptors [10], to a biological activity of interest through a mathematical/statistical approach. Throughout the years, many QSAR models have been proposed [11], based on different statistical techniques, such as Support Vector Machine (SVM) [12,13,14,15], *k*-Nearest Neighbors (*k*-NN) [16] and Self Organizing Maps (SOM) [17]. However, the majority of proposed QSAR models have some limitations that can often hamper the applicability and reliability of their predictions, especially for ADMET (adsorption, distribution, metabolism, excretion and toxicity) applications. In particular, the most notable drawbacks are (1) a limited applicability due to the small datasets they are often trained on or to the lack of an applicability domain assessment; and (2) the lack of biochemical interpretability due to the use of very complex modelling approaches and molecular descriptors.

The present work stems from these considerations and targets the development of novel, simple and easily interpretable QSAR systems to screen the drug binding to 3A4 and 2C9 isoform of CYP, which account for the metabolism of 30% and 13% of known drugs, respectively. Models were developed on High-Throughput Screening data and special attention was paid to model validation, applicability domain assessment and interpretation of the selected molecular descriptors.

## 2. Results and Discussion

### 2.1. Modelling Approach

**Figure 1:**
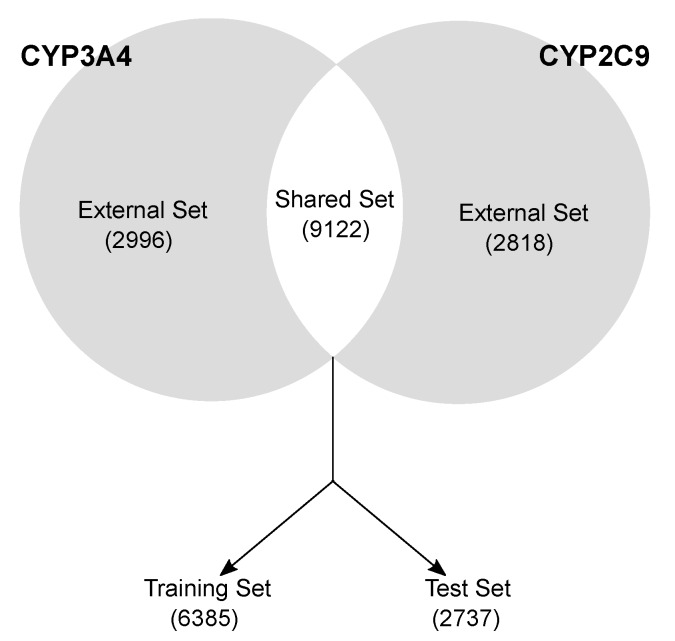
Scheme of the data splitting.

The starting point of this study was the database of potency values for 17,143 drug-like compounds for five CYP isoforms developed by Veith *et al.* [18], determined through quantitative High-Throughput Screening with a bioluminescent assay, which recognizes both inhibitors and substrates. The database was retrieved from PubChem (AID: 1851) [19]. Our modelling approach followed six logical steps:

1. Data curation and splitting. CYP3A4 and CYP2C9 isoforms were curated separately by removing mismatching duplicates, inconsistent records and disconnected structures. The molecules were divided in (a) a Shared set, comprising the 9122 compounds with annotated activity for both the isoforms (Figure 1) and (b) an External set, having activity data for one isoform molecules (2996 and 2818 for CYP3A4 and CYP2C9, respectively). The Shared set molecules were randomly split into a training (70%, 6385 compounds) and a test set (30%, 2737 compounds), keeping the active/inactive proportion of both the isoforms (49:100 and 66:100 for 2C9 and 3A4, respectively). The training set served to select the variables, calibrate the models and perform the cross-validation (five-fold). The test set was used only in a later stage to validate the final pool of selected models. The external sets were used in the final stage to further validate the best models.
2. Molecular description. To allow for the mathematical treatment of molecules, they were described using the so-called molecular descriptors [10], that is, numbers encoding for the presence of particular structural features, fragments or chemical properties. Two types of descriptors were calculated: (a) 3763 classical Dragon 6 [20] molecular descriptors (MDs) from 0-dimensional to 2-dimensional molecular representation, from which only a set of 1472 non redundant MDs was finally retained (see Materials and Methods); and (b) two types of binary fingerprints (FPs), that is, the extended connectivity (ECFP) [21] and the path fingerprints (PFP) [22], which are 1024 bit strings encoding the presence of particular fragments/substructures of molecules. Three-dimensional descriptors were not considered, as in a preliminary phase they did not lead to an improvement in the predictions.
3. Variable selection and modelling. The Genetic Algorithms (GA) [23], a benchmark variable selection method characterized by an optimal trade-off between computational time and exploration/exploitation ability [24], were used to retain the most relevant subsets of variables. A refined two-step GA procedure (see Materials and Methods) was applied on the training set descriptors in combination with six classification techniques: (a) Classification and Regression Trees (CART) [25]; (b) *k*-Nearest Neighbor (*k*-NN) [26]; (c) *N*-Nearest Neighbors (N3) [27]; (d) Binned Nearest Neighbors (BNN) [27]; (e) Linear Discriminant Analysis (LDA) [28]; and (f) Partial Least Squares Analysis (PLSDA) [29]. Note that for FPs, no variable selection was performed, as they, unlike MDs, give a description of the molecule when used as a whole. To model FPs, only similarity-based classifiers (*k*-NN, N3 and BNN) were used. On both the isoforms, the best results were obtained using: (1) CART [25], based on binary splits of the data using one variable at time according to its optimized threshold values; (2) *k*-NN [26], in which every molecule is classified according to the majority vote of its *k* more similar objects [14]; and (3) N3 [27], which uses all the available molecules as neighbors and, through an optimized α exponent, tunes their contribution as decreasing with decreasing their similarity to the new object. The model parameters (number of objects per leaf, *k* and α) were optimized in cross-validation as those giving the best classification performance.
4. Model selection and validation. From the pool of calculated models, the final models were chosen as the best compromise between classification performance in five-fold cross-validation (the higher the better) and number of variables (the smaller the better). Models with interpretable descriptors, if relevant, were preferred.
5. Applicability Domain Assessment. The selected models were evaluated for their chemical space of prediction reliability (Applicability Domain, AD). The AD assessment strongly depends on the nature of the modelling approach and the characteristics of the dataset [30], thus, it was calibrated it on a case-by-case basis, and rationalized according to the modeling approach (see Materials and Methods).
6. External validation. Models were selected according to the cross-validation results and the best models were screened on their performance on the test set. Finally, for each isoform, the external set molecules were used in order to test their robustness and predictivity towards real unknown data.

The model performance in recognizing active/inactive compounds was calculated through the Sensitivity (*Sn*), Specificity (*Sp*) and Non-Error Rate (*NER*), defined for two-class problems as follows:

(1) $$\begin{array}{c} Sn=\text{​ }\frac{TP}{TP+FN}\\ \begin{array}{l} Sp=\text{​ }\frac{TN}{TN+FP} \end{array}\\ NER=\text{​ }\frac{Sn+Sp}{2} \end{array}$$

where *TP*, *TN*, *FP* and *FN* are the number of true positives, true negatives, false positives and false negatives of each class, respectively. *Sn*, *Sp* and *NER* were calculated in fitting, cross-validation, and on the test/external sets.

### 2.2. Quantitative Structure-Activity Relationship (QSAR) Models

#### 2.2.1. Isoform 3A4

The proposed QSAR models for 3A4 are collected in Table 1. For all the models, a similar performance on the training and test sets can be noted, indicating the robustness and reliability of the predictions towards unknown data. The CART model, which is based on three very simple molecular descriptors, showed a very good balance between *Sn* and *Sp*. The *k*-NN model has a slightly better prediction ability, especially for the inactive compounds (higher *Sp*). If the model is restricted to its applicability domain (AD), more balanced predictions (in terms of *Sn* and *Sp*) are obtained with the same *NER* value. Finally, the N3 model (based on ECFPs) is characterized by high *Sn* values, that is, it identifies well the active compounds.

**Figure 2:**
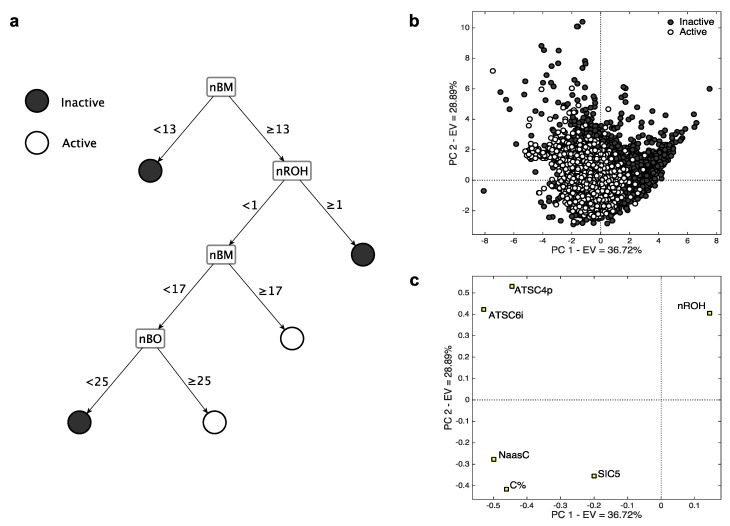
Representation of the molecular descriptor (MD)-based models for CYP3A4: (**a**) Classification and Regression Trees (CART) model; (**b**) Score plot of the training molecules described by the *k*-Nearest Neighbours (*k*-NN) descriptors, coloured according to their activity; (**c**) Loading plot of the *k*-NN descriptors.

The selected MDs are briefly presented in Table 2. In Figure 2a, their role in the CART classification is depicted. To visualize the effect of the descriptors on the *k*-NN model, a Principal Component Analysis (PCA) [31] was performed (Figure 2b,c). PCA is a well-known data visualization technique that allows to observe the objects in a few new variables (Principal Components, PC) space, according to their coordinates (scores, Figure 2b). The contribution of the variables in the PC space is given by the loadings (Figure 2c), the higher they are (in absolute value) the larger their role. PC1 and PC2 explain 2/3 of data variance. Information about PC3, which explains 18% of data variance, is reported in the Supplementary Material (Figure S1). PC3 explains 14% of data variance, and it leads to considerations similar to those derived from PC1/PC2. The PCA loadings (Figure 2c) allow for an understanding of the contribution of the molecular descriptors: the higher their PC1 loadings, the higher their value for inactive molecules and *vice* *versa* for active compounds. Active compounds group on the left side of the score plot (negative PC1 scores), while the inactive molecules distribute on the right side (positive PC1 scores). At the same time, active compounds have relatively lower PC2 values, indicating that high PC2 values relate to inactivity. The descriptors with high loadings on PC1 (e.g., *nROH*) and PC2 (e.g., *ATSC4p* and *ATSC6i*) influence at most the observed class separation. With the aid of the PCA and the CART classification scheme, a brief description and interpretation of the descriptors is given below.

1. The descriptor *nROH* represents the number of hydroxyl groups. It was independently selected in both the models based on MDs, underscoring its relevance in modelling the CYP3A4 activity. In particular, in both cases (Figure 2), high *nROH* values tend to correspond to inactive molecules. An increasing number of hydroxyl group generally increases the hydrophilicity, while molecules with no (or a few of) hydroxyl groups have a lipophilic nature. Molecular lipophilicity is known to facilitate the adsorption and to limit the excretion of the compounds. As a result, lipophilic molecules are oxidated by CYP and converted into more hydrophilic compounds that can be easily eliminated [32].
2. *nBM* and *nBO* are the number of multiple bonds and bonds in the H-depleted molecular structure, respectively, which were selected within the CART model (Figure 2c). The descriptor *nBM* represents a measure of the unsaturation level and, therefore, gives information about the molecular interaction ability with CYP. In addition, this descriptor contains information about molecular size, flexibility, presence of heteroatoms (Table 2). In particular, small and flexible molecules with a few of the multiple bonds (*nBM* < 13, Figure 2c) tend to be inactive. The relevance of heteroatoms can be related to the P450-mediated oxygen addition to nitrogen, sulfur, phosphorus, and iodine atoms [33,34,35,36].
3. The descriptor *nBO* mainly encodes the information about molecular size. Molecules with no hydroxyl groups, less than 16 multiple bonds and with less than 25 non-hydrogen bonds tend to be inactive. These molecules probably have a small effective dimension within the receptor pocket, as they are either small or relatively small, but very flexible. In this branch also lie some hydrophilic compounds classified as inactive. They probably do not interact with CYP as they are easily dissolved in the aqueous body fluid and, consequently, are excreted from the body [37].
4. *C%* and *NaasC* are the percentage of C atoms and number of aromatic carbons bonded with non-H atoms, respectively (Table 2). They have a crucial role in identifying the active compounds of *k*-NN, as shown by their very high loadings on PC1. This means that relative large and/or aromatic molecules are generally classified as actives. The mechanisms of aromatic oxidation have been already elucidated [36,38].
5. *SIC5* is the structural information content of order 5 [39]. It encodes information about atom equivalence and represents a general measure of structural complexity, the higher, the larger *SIC5*. It has negative PC1/PC2 loadings, suggesting that relatively large, branched and/or polycyclic compounds tend to be active. The effect of dimension on activity was already suggested (e.g., [40]), as well-known CYP3A4 ligands are commonly relatively large molecules.
6. *ATSC4p* and *ATSC6i* are the Centred Broto-Moreau autocorrelations [10] of lag 4 and 6, respectively. The former is weighed on the atomic polarizability, while the latter on the atomic ionization potential. They lie very close to each other in the PC space and have positive loadings on PC2, indicating that inactive compounds tend to have high values of both these MDs. They increase when increasing the molecular dimension, the number of heteroatoms and the branching/cyclicity. Because of their weighting scheme type, *ATSC4p* and *ATSC6i* increase when increasing the atomic polarizability and the ionization potential, respectively. These features are known for their relevant contribution in receptor binding, especially when charged systems are taken into consideration [41,42].

**Figure 3:**
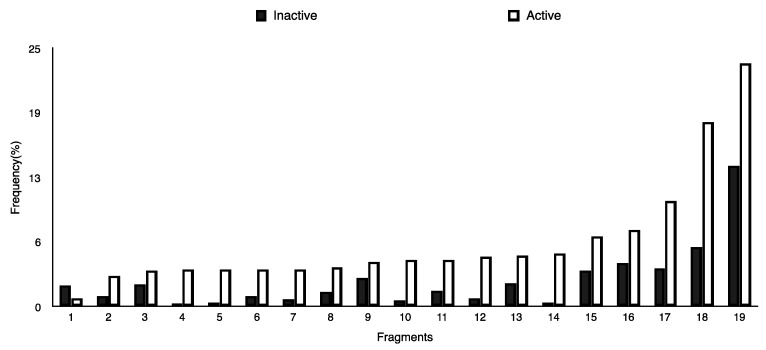

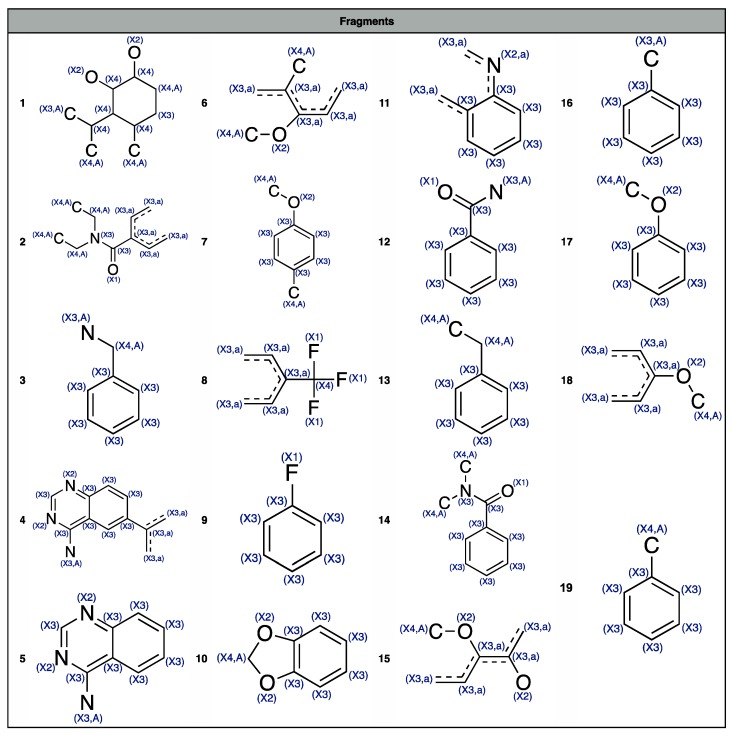
Occurrence frequency of the 19 selected fragments for CYP3A4 within the active/inactive compounds. Symbols associated with the fragments (according to SMARTS language) have the following meaning: *X* + number = number of total bonds in which the considered atom is involved; a = aromatic atom; A = aliphatic atom.

In order to interpret the ECFP, the relevant molecular fragments (MFs) encoded by ECFP were generated by means of Dragon 7 [22], with the same settings used for the fingerprints calculation. A small set of 19 large fragments (length from 4 to 6) was retained from the original pool by: (a) removing those that were not selected for at least 100 molecules; and (b) considering only those that were characterized by a difference of frequency between the classes greater than or equal to 1%. The fragments are depicted in Figure 3. Only the cyclohexanediol derivate fragment (No. 1) occurs more frequently within the inactive compounds. All the other MFs have a higher frequency for the active molecules. In particular, the MFs quinazolinamine-like (No. 4 and 5) and benzamide-like (No. 14) MFs are mostly present within active compounds (frequency ranging from 3.6% to 5.1%), and their frequency value within the inactive molecules is lower than 0.5%. The butenamide- (No. 2) and fluorobenzene (No. 9)-derived MFs are less useful for the discrimination between the classes, since they have the lowest difference in the frequency. The remaining fragments are high for both the classes.

#### 2.2.2. Isoform 2C9

The statistics of the selected classification models for the CYP2C9 are reported in Table 3. As for the 3A4 isoform, in this case the proposed models also have comparable performance on the training and test set. Furthermore, the CART model still appears to be the simplest and most balanced model, and only a few of the compounds are outside of its AD.

The best performance in recognizing inactive compounds was obtained with the *k*-NN approach (Table 3). On the contrary, N3 model, which is known for its ability to predict better the less represented class, is better in the actives recognition. The applicability domain characterization for the *k*-NN model slightly worsens the predictions on inactive compounds. For the N3 model, only a small fraction of compounds was excluded, not affecting the classification performance.

**Figure 4:**
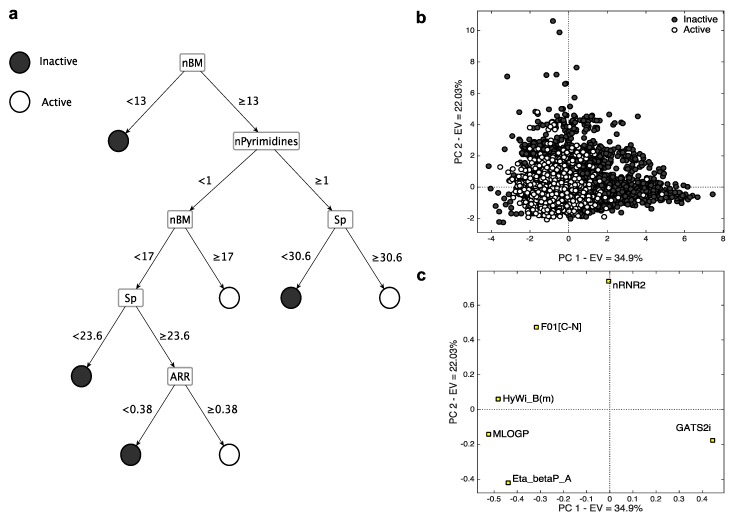
Representation of the MD-based models for CYP2C9: (**a**) CART model; (**b**) Score plot of the *k*-NN descriptors, colored according to their activity; (**c**) Loading plot of the *k*-NN descriptors.

Table 4 collects all the selected MDs for CYP2C9. In analogy with the previous case, in Figure 4, the representation of the CART model (Figure 4a) and of the PCA on *k*-NN descriptors (Figure 4b,c) can be found. In this case, PC1 and PC2 explain 57% of data variance. PC3 explains 14% of data variance and leads to similar considerations than those derived from PC1/PC2 (see Supplementary Material). In analogy with CYP3A4, the active compounds are grouped on the left part, having a low score value on PC1 and PC2, while the inactive compounds mainly cover the right side (high PC1 and PC2 scores).

The selected MDs are briefly described and interpreted below:

- *nBM* (number of multiple bonds) resulted to be relevant also for this isoform. As for the 3A4 CART model, small flexible molecules with a few number of multiple bonds (*nBM* < 13, Figure 4c) are classified as inactive, while those with a high number of multiple bonds (*nBM* > 17, Figure 4c) are classified as active.
- The presence of pyrimidines (*nPyrimidines*), together with the high values of *Sp* (sum of atomic carbon-scaled polarizability, *Sp* ≥ 30.6) characterize the active compounds. The inhibition ability of pyrimidine derivatives towards CYP2C9 was already suggested [44,45,46,47].
- *ARR* represents the aromatic ratio, that is, the ratio between the number of aromatic bonds and bonds in the H-depleted molecular structure. Molecules without pyrimidine rings but with an aromatic character (*ARR* ≥ 0.38) and high atomic polarizability value (*Sp* ≥ 23.6) are active. The presence of two MDs related to aromaticity (*i.e.*, *ARR* and *nPyrimidines*) suggests that this feature is fundamental for CYP2C9-drug interaction, in agreement with previous studies (e.g., [40]).
- *GATS2i* is the Geary autocorrelation of lag 2 weighted by the ionization potential [10]. It plays a crucial role in identifying the inactive compounds for the *k*-NN model, as denoted by its (high) positive loading on PC1. The inactive compounds, distributed on the right side of the score plot, are characterized by low values of this MD and have, therefore, low ionization potential. When increasing the ionization potential, the number of active compounds increases.
- *nRNR2* counts the number of aliphatic tertiary amines. It has high PC2 loading, meaning that it tends to be higher for inactive compounds. Tertiary aliphatic amines are, in fact, generally oxidized by Flavin monooxygenase [48] and are inactive on CYP.
- *F01[C–N]*, represents the frequency of bonded C and N atoms. It has a positive loading on PC2 and, in analogy with *nRNR2*, tends to be higher for inactive compounds, meaning that a high number of C-N could limit the interaction with the CYP2C9 isoform. In addition to the information overlap with *nRNR2*, *F01[C–N]* also takes into account the information about the presence of primary/secondary amines and of amides.
- *Eta_betaP_A*, is the average measure of π bonds and lone pairs of non-H atoms [49]. It has the lowest loading on PC2 and it increases when increasing the number of multiple bonds and lone pairs. Molecules with a high value of *Eta_betaP_A* tend to be active, confirming the previous considerations, that is, the higher the unsaturation, the higher the probability of activity towards CYP.
- *MLOGP* is the Moriguchi octanol-water partition coefficient [50]. Drug lipophilicity is known for playing a crucial role in the CYP binding affinity [51], and a significant correlation was reported between the P450 binding affinity and the compound lipophilicity [51,52,53,54]. As noted from the PCA, the compounds with high *MLOGP* value are active.
- *HyWi_B(m)* is the hyper-Wiener-like index weighted by mass [10]. It contains information about molecular size, branching, cyclicity and presence of heavy atoms. *HyWi_B(m)* tends to be higher for relatively large and polycyclic compounds, in analogy with the 3A4 isoform. As this descriptor captures several types of chemical information and its contribution is not easily detectable from the PCA, it needs further investigation.

**Figure 5:**
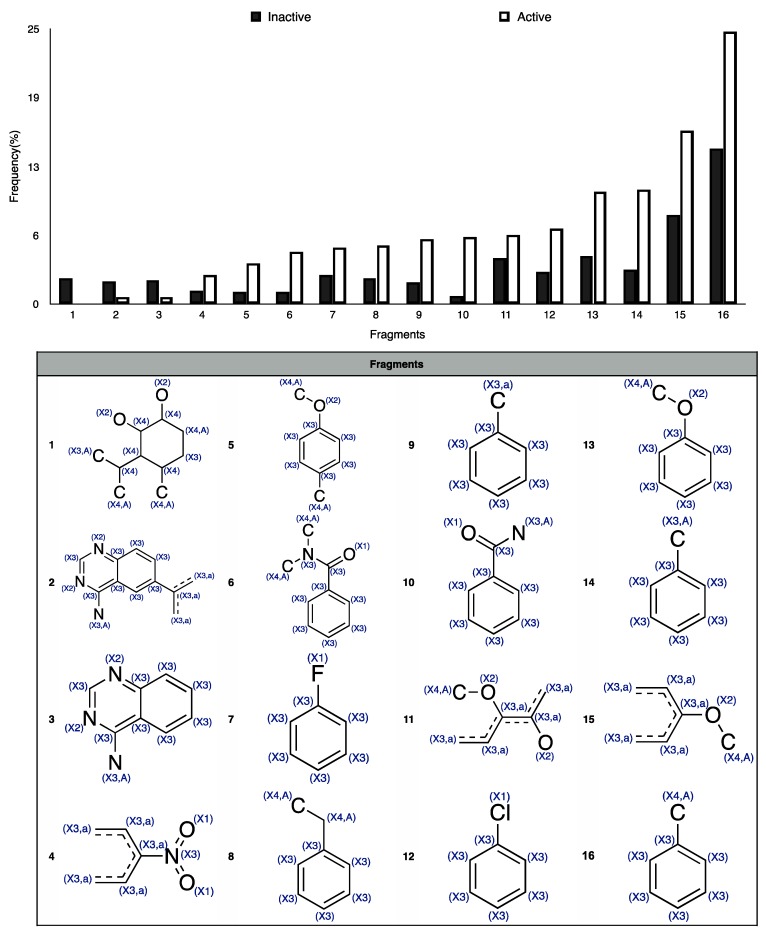
Occurrence frequency of the 16 selected fragments for CYP2C9 within the active/inactive compounds. Symbols associated with the fragments (according to SMARTS language) have the following meaning: *X* + number = number of total bonds in which the considered atom is involved; a = aromatic atom; A = aliphatic atom.

In order to easily interpret information encoded by CYP2C9 fingerprints, the same approach as for 3A4 was used (Figure 5), obtaining in this case a subset of the 16 most relevant molecular fragments (MFs). Also for this isoform, the cyclohexanediol-like MF (No. 1) mainly characterizes the inactive compounds. The quinazolinamine-like MFs (No. 2 and 3) have a higher frequency within inactive structures. This is a relevant difference with respect to the isoform, in which the opposite situation was observed. The remaining MFs are more frequent within the actives class, and the same conclusion as for 3A4 isoform can be drawn.

### 2.3. Consensus Modelling

The consensus approach is well-known in the field of QSAR [58,59,60,61] and consists in combining the predictions of several models to maximize their advantages and minimize their drawbacks, aiming to increase the global prediction accuracy. In this work, the selected classification models, which are based on very different descriptors and classification techniques, were merged in two types of *consensus* approaches (Table 5):

1. *Consensus* 1: a molecule was classified if and only if the following conditions were met: (1) all the model predictions agreed in its predicted class; (2) the molecule was inside the AD of all of the models.
2. *Consensus* 2: is based on the majority vote approach, *i.e.*, the compound is classified according to the most frequently predicted class. In this case, the AD of the models used for the prediction was considered.

For both the isoforms, the same outcome of the consensus approach was reached. Consensus 1 provided increased predictions reliability, especially for the active compounds (*Sn* ranging from 0.89 to 0.92 on the test set), at the expense of a large number of excluded molecules (up to 40% when both the AD and the model disagreement are considered). Consensus 2 showed a *NER* comparable with the single models, but with more balanced *Sn* and *Sp* values and the advantage of providing a prediction for each model.

### 2.4. External Validation

After recalibrating the models including the test set compounds within the AD domain, their predictivity was further tested on each isoform’s external set (Table 6). The single model performances tend to decrease on the external set. In particular, for CYP3A4 individual models, the *Sp* decreases substantially, while *Sn* values are stable; this means that the considered models are more reliable in recognizing the actives. This represents a prominent feature in the field of drug-drug interaction prediction and virtual screening [62,63]. On the contrary, on CYP2C9 single models, a comparable and moderately high decrease of *Sn* and *Sp* values can be noted, with the exception of N3, which has higher *NER* and *Sn* values. The best results are reached by the consensus 1 for both the isoforms, confirming the reliability of these models. For what concerns the consensus 2, they are slightly less accurate than the former case, but the advantage is that the predictions are provided for all the molecules, with a higher predictivity than the single models.

## 3. Materials and Methods

### 3.1. Data Curation

This study is based on the publicly available CYP bioactivity database developed by Veith *et al.* [18] of potency values for 17,143 drug-like compounds for five Cytochrome P450 isoforms (3A4, 2D6, 2C9, 2C19, 1A2), screened using a quantitative High-Throughput Screening with a bioluminescent assay, which recognizes both inhibitors and substrates. The dataset was retrieved from PubChem (AID: 1851), which provides the class of activity (active/inactive) for each compound, identified by a SMILES (Simplified Molecular Input Line Entry System). For each isoform of interest (3A4, 2C9), data were curated by: (a) removing the records without SMILES and/or activity class; (b) removing duplicate structures with mismatching class activity; (c) removing disconnected structures. The comparison between the isoform datasets led to the development of two types of sets (Table 7):

1. Shared set (9122 molecules), comprising the molecule available for both the isoforms. It served to calibrate, validate and choose the optimal model(s) for each isoform.
2. External sets (2996 and 2818 molecules for CYP3A4 and CYP2C9, respectively). These datasets comprise the molecules with annotated activity values for one isoform only. They were used as an external validation tool to further evaluate the model predictivity on unknown molecules.

The shared set of molecules was randomly split into a training set (70%, 6385 compounds) and a test set (30%, 2737 compounds), keeping the active/inactive proportion of both the isoforms (49:100 and 66:100 for 2C9 and 3A4, respectively). The training set was used to select the variables, calibrate the models and cross-validate them (five-fold). The test set served in a later stage to validate the final pool of selected models. The external set was used as a further validation tool only.

### 3.2. Molecular Descriptors

Two types of molecular descriptors were considered:

1. Classical molecular descriptors (MDs) were calculated using Dragon 6 [20]. From the obtained 3763 MDs, we filtered out those that: (a) were constant or near-constant; (b) had at least one missing value; (c) had a pairwise correlation larger than 0.95. Eventually, 1472 descriptors were retained.
2. Two types of binary fingerprints (FPs) were calculated, namely: (1) extended connectivity (ECFP) [21], circular/topological FPs that encode for several molecular features including stereochemical information; and (2) path connectivity (PFP) [64] based on the presence of particular molecular fragments without accounting for the stereochemical information. A total of 1024 bit FPs were calculated using Dragon 7 [22]. The detailed settings can be found in the Supplementary Material. PFP were considered in the preliminary phase, but their results were not reported as they were outperformed by ECFP.

### 3.3. Variable Selection

The most relevant MDs were selected using Genetic Algorithms [23], in the version proposed by Leardi *et al.* [65]. For each method, the following strategy was always followed: (1) GA were run on the pool of MDs (1472); (2) the most frequent MDs of phase 1 (up to a maximum of 200) were subjected to a second selection phase; and (3) the most relevant MDs of phase 2 (up to 15) were tested for all of their possible combinations (All Subset Modelling Strategy, e.g., [66]).

### 3.4. Applicability Domain (AD) Assessment

The AD approach was rationalized according to the classifier characteristics, as explained below.

1. As CART is based on univariate splits of data, a “bounding box” approach was applied, by excluding the molecules with descriptor values outside the training set ranges (reported as Supplementary Material) [58].
2. For *k-*NN, every compound too far from its *k* neighbors was considered out of the AD, using the approach proposed by Sahigara *et al.* [67]. The same distance metric of the best *k*-NN models (Euclidean distance) was used.
3. For what concerns N3, as the largest contribute to the classification is given by the closest compound, a query was considered inside the AD if its similarity with its nearest neighbor was larger than or equal to 0.2. The same similarity metric of best models (Jaccard-Tanimoto) was used.

### 3.5. Software and Code

Data curation was performed using KNIME [68] v 2.11.3 workflows. Training/test splitting, variable selection, cross-validation and model calibration were performed in MATLAB [69] environment, using routines written by Milano Chemometrics and QSAR Research Group [70]. 

## 4. Conclusions

The overall goal of this work was to obtain reliable QSAR screening systems for two of the most relevant isoforms of Cytochrome P450, namely CYP2C9 and CYP3A4.

The study was based on the CYP bioactivity database developed by the National Institutes of Health Chemical Genomics Center (NCGC), from which activity data for 14,936 molecules were obtained and used to train/validate the models.

Different classification approaches were tested on both classical molecular descriptors and binary fingerprints. For each isoform, three models led to the optimal results: (1) a classification tree, characterized by high interpretability and prediction balance on the classes; (2) a *k-*Nearest Neighbor model, with high complexity but also high predictivity towards inactive compounds; and (3) a *N*-Nearest Neighbor model, which had the highest performance towards the actives.

The interpretation of the selected molecular descriptors made it possible to gather insights into the structural features that determine the activity on Cytochrome P450. In particular, for both the isoforms, molecular dimension and flexibility ultimately influenced the activity towards CYP, in that small and flexible molecules tend to be inactive, while lipophilic compounds tend to be active. As for CYP3A4, distinctive features of the active molecules turned out to be the presence of hydroxyl groups, and/or a low polarizability/ionization potential. Regarding the CYP2C9, the analysis underscored the fact that active molecules tend to have a large number of aromatic bonds, along with a high polarizability, a high unsaturation degree and often the presence of pyrimidines. Moreover, the interaction with CYP2C9 could be limited by a high number of bonded C and N atoms in the molecule.

As a final classification step, the models were aggregated in a *consensus* manner. This allowed us to obtain a system with a good predictive ability towards both the activity classes and to exploit the advantages of the single models. Finally, the models were validated using more than 2000 molecules for each isoform as an external set. The external validation confirmed the model reliability and stability. A future perspective will be the development of analogous approaches for the remaining CYP isoforms of relevance.

## Figures and Tables

**Table 1:** Model statistics for CYP3A4 isoform. Models are described according to the method and type of descriptors, the Applicability Domain (AD: yes/no (y/n)), number of variables (*p*) and classification parameters (parameter: object/leaf ratio for Classification and Regression Trees (CART), *k* for *k*-Nearest Neighbours (*k*-NN) and α for *N*-Nearest Neighbors (N3)). For each model, the Non-Error Rate (*NER*), the Sensitivity (*Sn*) and the Specificity (*Sp*) are reported in Fitting, Cross-Validation and on the test set. %out indicates the percentage of test set compounds outside of the AD. MD: molecular descriptors; ECFP: extended connectivity fingerprints.

Model	Descriptors	AD	*p*	Parameter	Fitting			Cross-Validation			Test Set			
					*NER*	*Sn*	*Sp*	*NER*	*Sn*	*Sp*	%out	*NER*	*Sn*	*Sp*
CART	MD	y	3	210	0.74	0.74	0.75	0.74	0.73	0.75	-	0.75	0.74	0.76
		n	3	210	0.74	0.74	0.75	0.74	0.73	0.75	0	0.75	0.74	0.76
*k-*NN	MD	y	6	14	0.76	0.73	0.79	0.76	0.73	0.78	-	0.77	0.75	0.79
		n	6	14	0.76	0.73	0.79	0.76	0.73	0.78	5	0.77	0.76	0.78
N3	ECFP	y	1024	1	0.79	0.88	0.71	0.79	0.87	0.70	-	0.78	0.86	0.71
		n	1024	1	0.79	0.88	0.71	0.79	0.87	0.70	1	0.78	0.86	0.71

**Table 2:** List and brief description of the classical molecular descriptors (MDs) selected for CYP3A4. Some examples of molecules with low and high MD values are also reported.

MD	Description	Reference	Model	Low Value		High Value	
*nBM*	Number of multiple bonds.	[10]	CART	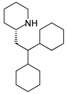	0	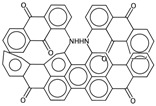	66
*nBO*	Number of non-hydrogen bonds.	[10]	CART	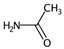	3	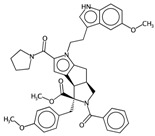	59
*C%*	Percentage of C atoms.	[10]	*k-*NN	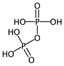	0	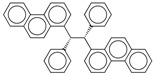	58.3
*SIC5*	Structural Information Content—order 5.	[39]	*k-*NN	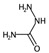	0.28	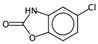	1.00
*ATSC4p*	Centred Broto-Moreau autocorrelations—lag 4 (weighted by atomic polarizability).	[20]	*k-*NN	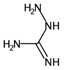	0.21	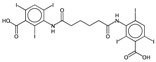	46.6
*ATSC6i*	Centred Broto-Moreau autocorrelations—lag 6 (weighted by atomic ionization potential).	[20]	*k-*NN	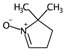	0	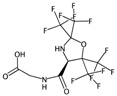	4.1
*nROH*	Number of hydroxyl groups.	[10]	CART, *k-*NN	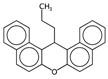	0	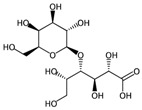	9
*NaasC*	Counts of the E-state atom types.	[43]	*k-*NN	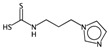	0	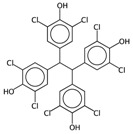	16

**Table 3:** Model statistics for CYP2C9 isoform. Models are described according to the method and type of descriptors, the Applicability Domain (AD: yes/no), number of variables (p) and classification parameters (parameter: object/leaf ratio for CART, *k* for kNN and α for N3). For each model, the Non-Error Rate (*NER*), the Sensitivity (*Sn*), and the Specificity (*Sp*) are reported in Fitting, Cross-Validation and on the test set. %out indicates the percentage of test set compounds outside the AD.

Model	Descriptors	AD	*p*	Parameter	Fitting			Cross-Validation			Test Set			
					*NER*	*Sn*	*Sp*	*NER*	*Sn*	*Sp*	%out	*NER*	*Sn*	*Sp*
CART	MD	y	4	210	0.75	0.75	0.75	0.75	0.75	0.75	-	0.75	0.75	0.74
		n	4	210	0.75	0.75	0.75	0.75	0.75	0.75	0	0.75	0.75	0.74
*k-*NN	MD	y	6	14	0.77	0.69	0.85	0.77	0.68	0.85	-	0.76	0.67	0.86
		n	6	14	0.77	0.69	0.85	0.77	0.68	0.85	5	0.76	0.67	0.84
N3	ECFP	y	1024	1	0.80	0.87	0.73	0.80	0.86	0.73	-	0.78	0.83	0.73
		n	1024	1	0.80	0.87	0.73	0.80	0.86	0.73	1	0.78	0.83	0.73

**Table 4:** List and brief description of the classical molecular descriptors (MDs) selected for CYP2C9. Some examples of molecules with low and high MD values are also reported.

MD	Description	Reference	Model	Low Value		High Value	
*nBM*	Number of multiple bonds.	[10]	CART	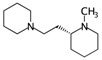	0	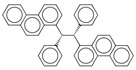	44
*Sp*	Sum of atomic polarizabilities scaled on Carbon atom.	[10]	CART	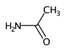	5.0	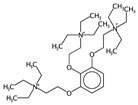	56.1
*ARR*	Ratio between the number of aromatic bonds over the total number of non-H bonds.	[10]	CART	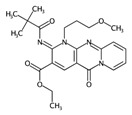	0	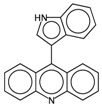	0.96
*HyWi_B(m)*	Hyper-Wiener-like index from Burden matrix weighted by mass.	[55]	*k-*NN	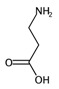	2.3	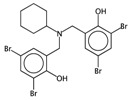	5.1
*GATS2i*	Geary autocorrelation of lag 2 weighted by ionization potential.	[56]	*k-*NN	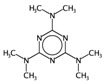	0.09	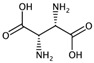	1.93
*Eta_betaP_A*	Eta pi and lone pair average VEM count.	[49]	*k-*NN	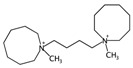	0	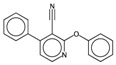	1.02
*nPyrimidines*	Number of Pyrimidines.	[10]	CART	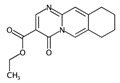	0	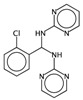	2
*nRNR2*	Number of aliphatic tertiary amines.	[10]	*k-*NN	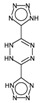	0	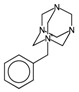	3
*F01[C–N]*	Frequency of C–N at topological distance 1.	[57]	*k-*NN	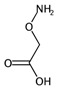	0	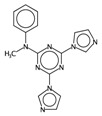	19
*MLOGP*	Moriguchi octanol-water partition coefficient.	[50]	*k-*NN	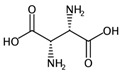	-6.3	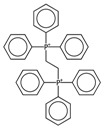	9.6

**Table 5:** *Consensus* models (*cons*) 3A4 and 2C9. Non-Error Rate (*NER*), Sensitivity (*Sn*), and Specificity (*Sp*) are reported in Fitting, Cross-Validation and on the test set.

CYP	Type	Fitting				Cross-Validation				Test set			
		%na	*NER*	*Sn*	*Sp*	%na	*NER*	*Sn*	*Sp*	%na	*NER*	*Sn*	*Sp*
3A4	*cons 1*	33	0.88	0.92	0.84	33	0.88	0.92	0.84	36	0.88	0.92	0.83
	*cons 2*	-	0.79	0.80	0.78	-	0.78	0.79	0.77	6	0.80	0.81	0.80
2C9	*cons 1*	33	0.89	0.90	0.88	34	0.89	0.90	0.88	40	0.89	0.89	0.88
	*cons 2*	-	0.81	0.80	0.82	-	0.81	0.80	0.82	1	0.79	0.77	0.81

**Table 6:** Classification results of the models on the external set in terms of *Sn*, *Sp*, *NER* and percentage of not assigned/out of the AD compounds (%na).

CYP	Mod.	Fitting ^a^				Cross-Validation ^a^				External Set			
		%na	*NER*	*Sn*	*Sp*	%na	*NER*	*Sn*	*Sp*	%na	*NER*	*Sn*	*Sp*
3A4	CART	-	0.75	0.74	0.75	-	0.74	0.73	0.76	-	0.66	0.68	0.63
	*k*-NN	-	0.76	0.73	0.79	-	0.76	0.73	0.79	1	0.70	0.70	0.69
	N3	-	0.80	0.87	0.73	-	0.79	0.87	0.71	1	0.72	0.85	0.59
	*cons 1*	32	0.88	0.91	0.85	33	0.88	0.91	0.84	42	0.80	0.89	0.70
	*cons 2*	-	0.80	0.80	0.79	-	0.79	0.80	0.78	1	0.71	0.76	0.67
2C9	CART	-	0.75	0.77	0.74	-	0.74	0.73	0.76	-	0.66	0.66	0.66
	*k*-NN	-	0.77	0.69	0.85	-	0.77	0.68	0.85	1	0.69	0.58	0.81
	N3	-	0.80	0.86	0.74	-	0.79	0.85	0.74	-	0.75	0.83	0.68
	*cons 1*	34	0.89	0.91	0.88	35	0.89	0.90	0.88	45	0.83	0.85	0.82
	*cons 2*	-	0.81	0.81	0.82	-	0.80	0.79	0.82	1	0.73	0.71	0.75

**^a^** Performance on the models recalibrated on all the shared set (9122 molecules), *i.e.*, on training and test set compounds.

**Table 7:** Characteristics of the shared and external sets for each isoform: *n* = number of molecules, %act = percentage of active compounds.

CYP Isoform	Shared Set		External Set	
	*n*	%act	*n*	%act
**3A4**	9122	40	2996	49
**2C9**	9122	33	2818	36

## Supplementary Materials

The curated datasets along with the calculated molecular descriptors and other supplementary materials can be found at http://www.mdpi.com/1422-0067/17/6/914/s1. The dataset can be also freely downloaded from Milano Chemometrics website (http://michem.disat.unimib.it/chm/).

## Abbreviations

AD	Applicability Domain
ADMET	Absorption, Distribution, Metabolism, Excretion and Toxicity
CART	Classification and Regression Trees
CYP	Cytochrome P450
FP	binary Fingerprints
ECFP	Extended Connectivity Fingerprints
*k*-NN	*k*-Nearest Neighbors
MD	classical Molecular Descriptors
MF	Molecular Fragment
N3	*N*-Nearest neighbors
*NER*	Non-Error Rate
QSAR	Quantitative Structure-Activity Relationship
SMILES	Simplified Molecular Input Line Entry System
*Sn*	Sensitivity
*Sp*	Specificity
