# Supplementary material for: In Silico Prediction of Cytochrome P450-Drug Interaction: QSARs for CYP3A4 and CYP2C9
Source: Int J Mol Sci. 2016 Jun 9;17(6):914. doi: 10.3390/ijms17060914 (PMC4926447; doi:10.3390/ijms17060914)
Supplement: Supplementary file 1 [file ijms-17-00914-s001.pdf]

Supplementary Materials: *In Silico* Prediction of Cytochrome P450-Drug Interaction: QSARs for CYP3A4 and CYP2C9

Serena Nembri, Francesca Grisoni, Viviana Consonni and Roberto Todeschini

1. Setting for Calculating the Extended Connectivity Fingerprints (ECFP)

Table S1. Settings used for calculation of the Extended Connectivity Fingerprints: size, bits per pattern, minimum and maximum length of fragments and atom features taken into account.

Size	Bits Per Pattern	Minimum Length	Maximum Length	Atom Options
1024	2	0	6	Atom type, Aromaticity, Connectivity (total), Charge, Bond order.

2. Classification and Regression Trees (CART), Models Calibration Settings

Table S2. Settings used for the Classification and Regression Trees (CART) models calibration for both the isoforms: object/leaf ratio, cost matrix and splitting criterion. CYP: Cytochromes P450.

CYP Isoform	Object/Leaf Ratio	Cost Matrix	Splitting Criterion
**3A4**	210	[0 0; 0 0]	Gini diversity index
**2C9**	210	[0 0.35; 0.65 0]	Gini diversity index

3. Applicability Domain Cart Models

Table S3. For each isoform, the minimum and the maximum values assumed by each Molecular Descriptors are reported included in the CART model. MDs: molecular descriptors.

CYP Isoform	MD	Range	
		min	max
**3A4**	*nBO*	3	93
	nBM	0	66
	nROH	0	12
**2C9**	*Sp*	8.960	90.318
	nBM	0	66
	ARR	0	0.963
	nPyrimidines	0	2

4. Modelling Methods Tested

Table S4 shows the best model for each of the classification methods tested: CART, *k*-Nearest Neighbours (*k*-NN), Linear Discriminant Analysis (LDA), *N*-Nearest Neighbors (N3) and Binned Nearest Neighbors (BNN).

Table S4. Model statistics for both the isoforms. Models are described according to the method, number of variables (*p*) and classification parameters (object/leaf ratio for CART, *k* for *k*-Nearest Neighbours (*k*-NN), latent variable for LDA and α for N3 and BNN). For each model, the Non-Error Rate (*NER*), the Sensitivity (*Sn*), and the Specificity (*Sp*) are reported in fitting, cross-validation and on the test set. ECFP: extended connectivity fingerprints.

CYP Isoform	Mod.	Descriptors	*p*	Parameters	Fitting			CV			Test		
					NER	*Sn*	*Sp*	*NER*	*Sn*	*Sp*	*NER*	*Sn*	*Sp*
**3A4**	CART	MD	3	210	0.74	0.74	0.75	0.74	0.73	0.75	0.75	0.74	0.76
	k-NN	MD	6	14	0.76	0.73	0.79	0.76	0.73	0.78	0.77	0.75	0.79
	N3	ECFP	1024	1	0.79	0.88	0.71	0.79	0.87	0.70	0.78	0.86	0.71
	LDA	MD	4	2	0.76	0.66	0.85	0.76	0.67	0.86	0.77	0.67	0.86
	k-NN	ECFP	1024	10	0.78	0.85	0.72	0.78	0.84	0.73	0.77	0.83	0.71
	BNN	ECFP	1024	0.35	0.79	0.80	0.78	0.79	0.80	0.78	0.77	0.78	0.77
**2C9**	CART	MD	4	210	0.75	0.75	0.75	0.75	0.75	0.75	0.75	0.75	0.74
	k-NN	MD	6	14	0.77	0.69	0.85	0.77	0.68	0.85	0.76	0.67	0.86
	N3	ECFP	1024	1	0.80	0.87	0.73	0.80	0.86	0.73	0.78	0.83	0.73
	LDA	MD	6	2	0.72	0.71	0.73	0.76	0.75	0.76	0.73	0.71	0.75
	k-NN	ECFP	1024	6	0.76	0.89	0.63	0.76	0.88	0.63	0.73	0.87	0.59
	BNN	ECFP	1024	1.15	0.77	0.87	0.66	0.77	0.87	0.66	0.75	0.86	0.64

5. Principal Component Analysis (PC1/PC3)

5.1. CYP3A4

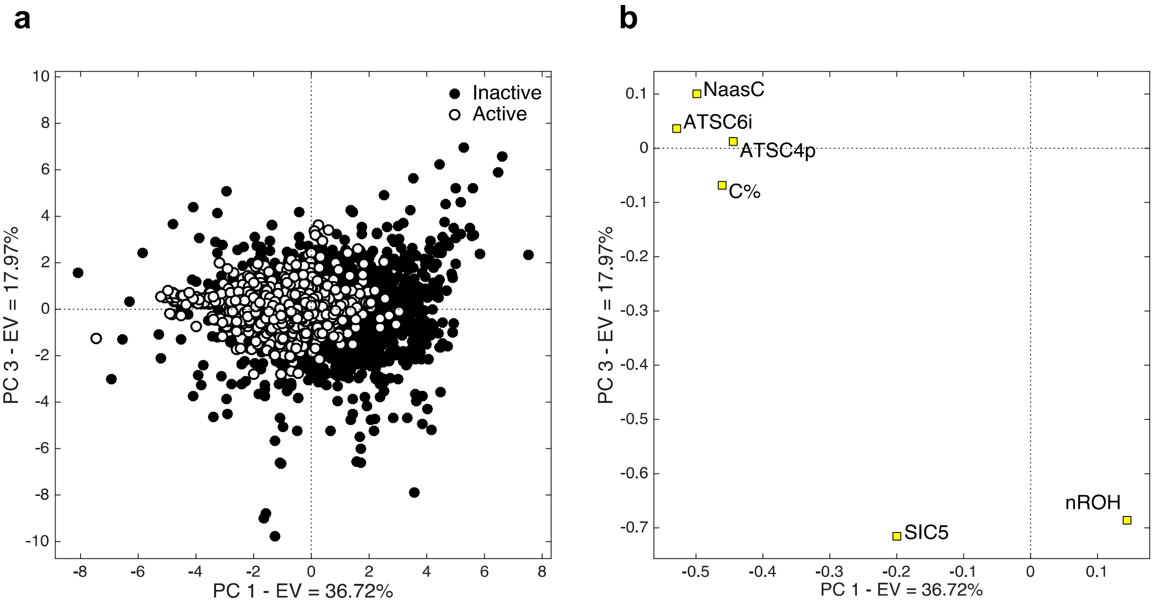

Figure S1. PCA based on PC1 and PC3 for CYP3A4: (**a**) Score plot of the training molecules described by the *k*-Nearest Neighbours (*k*-NN) descriptors, coloured according to their activity; (**b**) Loading plot of the *k*-NN descriptors.

5.2. CYP2C9

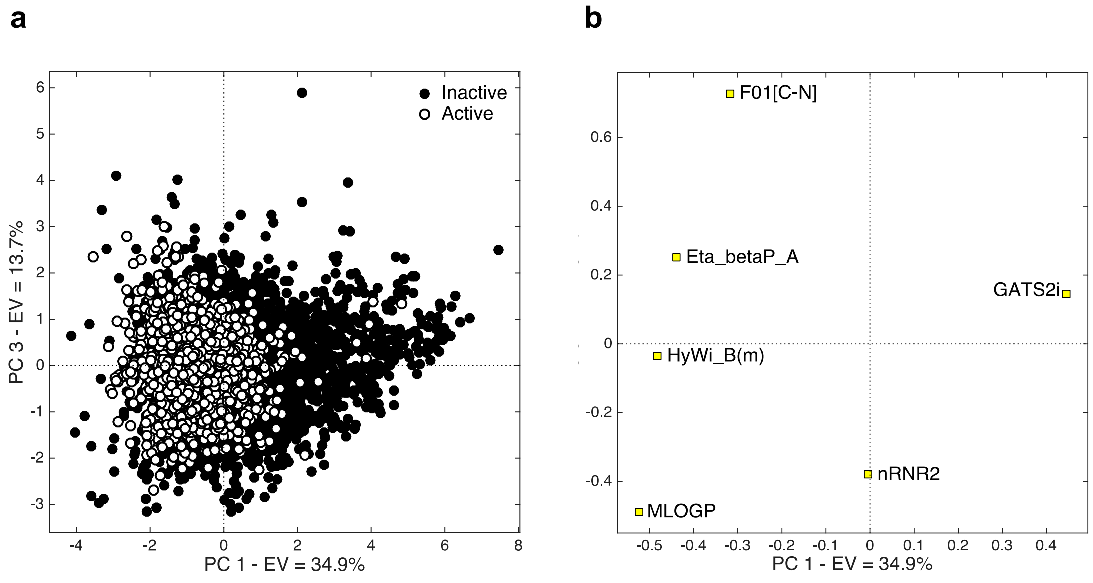

Figure S2. PCA based on PC1 and PC3 for CYP2C9: (**a**) Score plot of the training molecules described by the *k*-NN descriptors, coloured according to their activity; (**b**) Loading plot of the *k*-NN descriptors.
